# Colorectal Cancer Risk and Protective Factors Among People of African Descent: A Systematic Review and Meta-Analysis

**DOI:** 10.7759/cureus.88791

**Published:** 2025-07-26

**Authors:** Collins C Okeke, Angela Ojo, Onyinye E. Ebiliekwe, Kris N Idion, Ifunanya R Ekeocha, Sylvahelen Okorienta, Afamefuna O Onyeogulu, Onyeka Egemonye, Ginika S Okafor, Oyiyechukwu R Charles, Chidera C Okoye, Euodia A Ugo-Ihanetu, Chinemerem Ukoha, Amarachi Nduji, Joseph O Egbunike, Omosimisola O Alli

**Affiliations:** 1 Internal Medicine, University of Port Harcourt Teaching Hospital, Port Harcourt, NGA; 2 Internal Medicine, Afe Babalola University Ado-Ekiti, Ado Ekiti, NGA; 3 Internal Medicine, Nnamdi Azikiwe University Teaching Hospital, Nnewi, NGA; 4 Internal Medicine, Delta State University Teaching Hospital, Oghara, NGA; 5 Public Health, Liberty University, Lynchburg, USA; 6 Internal Medicine, Nnamdi Azikiwe University College of Health Sciences, Awka, NGA; 7 Internal Medicine, Federal Teaching Hospital, Ido-Ekiti, NGA; 8 Hematology, Delta State University Teaching Hospital, Oghara, NGA; 9 Internal Medicine, Chukwuemeka Odumegwu Ojukwu University Teaching Hospital, Awka, NGA; 10 Internal Medicine, Rivers State University Teaching Hospital, Port Harcourt, NGA; 11 Internal Medicine, University of Abuja Teaching Hospital, Abuja, NGA; 12 Internal Medicine, Abia State University Uturu, Uturu, NGA; 13 Family Medicine, Aeon Medical Center, Abuja, NGA; 14 General Practice, Lagos State University College of Medicine, Lagos, NGA

**Keywords:** african, african american, african descent, colorectal cancer, protective factors, risk factors

## Abstract

Colorectal cancer (CRC) remains one of the leading causes of cancer-related morbidity and mortality worldwide, particularly affecting populations in low- and middle-income countries. Lower screening utilization among African descent has been linked to delayed diagnosis and early intervention. This review aims to identify the most common risk and protective factors of CRC among people of African descent. A comprehensive search was conducted on PubMed and Google Scholar databases from inception to the 21st of April 2025, and 5062 articles were synthesized. Of these, 30 articles were included for final qualitative analysis, data extraction, and meta-analysis. We included original peer-reviewed articles written in the English language that report CRC patients' possible risk or protective factors for CRC among adult patients of any gender and of African descent in any country around the world. Our review included 53,596 people of African descent across nine countries, and 8317 (16%) of them were diagnosed with CRC via colonoscopy and histology. The non-modifiable risk factors reported include: age > 55 years, family history of CRC, male gender, family history of malignancy, and genetic mutation, while the modifiable risk factors include: tobacco smoking, frequent consumption of alcohol, red/processed/roasted meat (suya), a fat diet, BMI > 30 kg/m², *Helicobacter pylori* infection, carcinogen exposure at work, and urban residence. Among the protective factors against CRC, frequent consumption of vegetables, fruits, fish, traditional African diet, nonsteroidal anti-inflammatory drug (NSAID) use, and physical activity were mostly reported. The pooled prevalence of CRC was 41.8% (95% CI: 39.6-44.1%), reflecting data primarily from hospital-based and high-risk populations, and not general population screening. Significant heterogeneity was observed (Cochran's Q > 1000, I² > 98%), indicating substantial variation among studies. Family history of malignancy, tobacco use, alcohol intake, obesity, and red or processed meat consumption emerged as the most consistently reported risk factors. Protective behaviors, such as high vegetable intake, physical activity, and adherence to traditional African diets, were underreported but suggest actionable avenues for prevention. The findings emphasize the urgent need for earlier and more culturally tailored screening interventions, as well as investment in molecular and registry-based research within African health systems.

## Introduction and background

Colorectal cancer (CRC) is a malignancy that develops from the tissues of the colon or rectum, which are part of the digestive system [1]. It ranks as the third most prevalent cancer worldwide and accounts for 10% of all cancer cases and the second leading cause of cancer-related mortality worldwide [2]. According to the WHO, about 1.9 million new cases of CRC and more than 930,000 deaths due to CRC were estimated in 2020. It is projected that by 2040, the burden of CRC will increase to 2.3 million new cases and 1.6 million deaths yearly [2]. In 2022, about 70,428 new cases and 46,087 deaths due to CRC were recorded across the African continent, with the highest in North and East Africa. In 2050, it is projected that the incidence of CRC in Africa will increase to 168,683 and 117,568 deaths [3]. Among all racial and ethnic groups, African-Americans experience the highest age-adjusted CRC incidence and mortality [4]. Lower screening programs and utilization, especially in the African continent, have been linked to delayed CRC diagnosis, which may help explain the observed disparities [4]. CRC remains one of the leading causes of cancer-related morbidity and mortality, particularly affecting populations in low- and middle-income countries [5]. The highest incidence rates of CRC are reported in regions such as Australia, New Zealand, Europe, and North America, whereas Africa and South-Central Asia have the lowest rates [6]. People of African descent live in many countries around the world, whether descendants of those Africans who were displaced to America during the transatlantic slave trade many generations ago, or more recent migrants who journeyed to America, Europe, Asia, and within Africa. The largest population of African descent can be found in America and the Caribbean, with an estimated 150 million. About 200 million people identify themselves as being of African descent in America, and many millions live in other parts of the world outside of the African continent [7].

The pathogenesis of CRC is multifactorial; the epithelial cells of the colorectal mucosa can undergo hyperplasia, atypical hyperplasia, and adenomas that eventually transform into a carcinoma. CRC can develop due to chromosomal instability that occurs mainly at familial adenomatous polyposis (FAP), genetic mutations, and hypermethylation at specific gene promoter regions [8]. CRC spreads through local invasion, lymphatic, hematogenous, and implantation metastasis. CRC can manifest with a variety of clinical features, exhibiting a diverse constellation of signs and symptoms that vary significantly depending on the tumor's precise anatomical location within the colon or rectum, its dimensions and extent of local invasion, the stage of disease progression, and the patient's individual physiological characteristics. The presenting symptoms can range from subtle alterations in bowel habits, such as persistent diarrhea or constipation, or changes in stool caliber, to more alarming signs like rectal bleeding, abdominal pain, unexplained weight loss, and fatigue The insidious nature of CRC often contributes to delayed diagnosis, as initial symptoms may be subtle, sporadic, and easily mistaken for more benign gastrointestinal disorders, including irritable bowel syndrome or hemorrhoids, potentially delaying timely medical intervention and comprehensive evaluation [2]. Some screening tests are beneficial in the early diagnosis and prevention of CRC. There are three main types of CRC screening tests: stool-based tests (fecal immunochemical test, guaiac-based fecal occult blood, multitargeted stool DNA or RNA test), visual exams (colonoscopy, sigmoidoscopy, CT colonography), and blood-based tests (Shield, ColoHealth) [9]. The earliest stage of CRC is stage 0 and ranges from stage I through IV [10]. The histological types of CRC include papillary adenocarcinoma, tubular adenocarcinoma, mucinous adenocarcinoma, signet-ring cell carcinoma, undifferentiated carcinoma, adenosquamous carcinoma, squamous cell carcinoma, and carcinoid carcinoma [8]. This review aims to identify the most common risk and protective factors of CRC among people of African descent.

## Review

This systematic review was conducted following the Preferred Reporting Items for Systematic Reviews and Meta-Analyses (PRISMA) extension for systematic reviews. The study protocol was registered with PROSPERO CRD420251039447.

Inclusion criteria

We included original peer-reviewed articles written in the English language that report CRC patients' possible risk or protective factors for CRC among adult patients of any gender and of African descent in any country around the world.

Exclusion criteria

We excluded abstracts, reports, comments, surveys, case reports, case series, editorial, systematic review, meta-analysis, patient < 18 years of age, non-English articles, presence of other cancer, studies reporting risk factors among multi ethnic groups, and studies excluding patients of African descent.

A search was done from inception to the 21st of April 2025, on PubMed and Google Scholar databases, with the following search phrases across the databases, ((risk factor) AND (colorectal cancer)) AND (African), ((risk factor) AND (colorectal cancer)) AND (African American), ((risk factor) AND (colorectal cancer)) AND (African Caribbean), ((risk factor) AND (colorectal cancer)) AND (Blacks), ((risk factor) AND (colorectal cancer)) AND (African European), "colorectal cancer" "African Descent", "colorectal cancer" "African American”, "colorectal cancer" "Africa". More details of the search strategy are shown in Appendix A. 

The search results from the systematic search of various databases were imported into Rayyan (Rayyan Systems Inc., Cambridge, MA) referencing manager [11], where duplicate, title, and abstract screening were carried out by three independent co-authors. Following abstract screening, the eligible articles were subjected to full-text screening using the predefined eligibility criteria. Disagreements were discussed among the authors, and another author was invited in case no resolution was reached. 

Data extraction from the eligible articles was conducted by four co-authors and was imputed independently into the Google spreadsheet. The extracted baseline variables include: author's name, country, study year, study design, sample size, population of interest, gender, mean age, body mass index (BMI), diagnostic tool, cancer location, educational status, and risk and protective factors. The eligible articles underwent quality assessment using the Joanna Briggs Institute (JBI) risk of bias critical appraisal tool for case-control and cohort studies. The purpose of this appraisal was to assess the methodological quality of the study and to determine the extent to which the study addressed the possibility of bias in its design, conduct, and analysis. Articles were assessed with a yes, no, not clear, and not applicable [12], as shown in Appendix B and Appendix C.

Results

**Figure 1:**
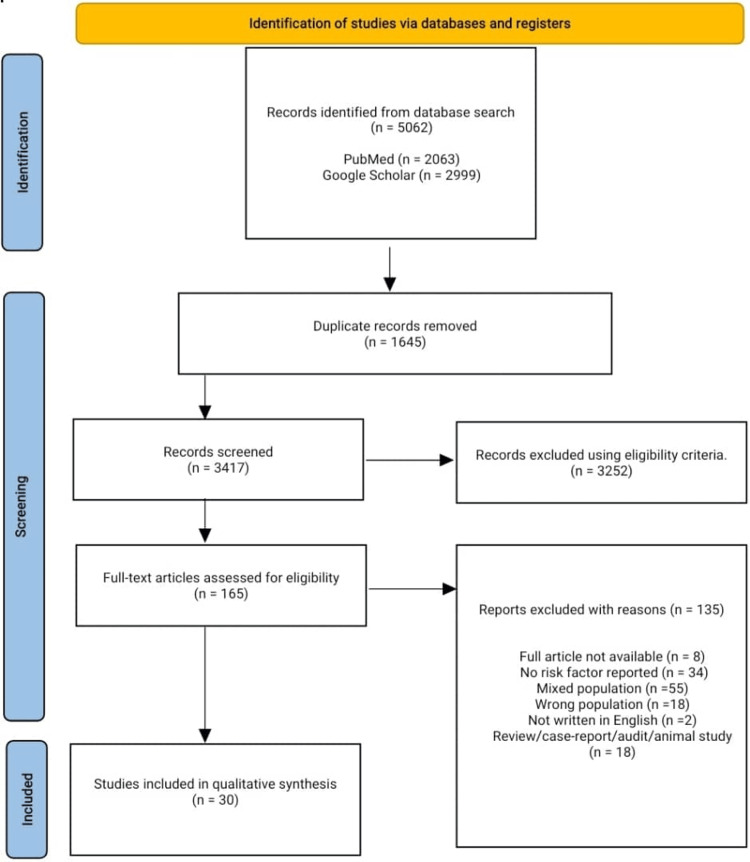
PRISMA flow diagram A methodological flow diagram following the RRISMA guideline for systematic reviews PRISMA: Preferred Reporting Items for Systematic Reviews and Meta-Analyses

Our systematic search across the databases yielded 5062 articles, 1645 of which were duplicates; 3417 articles were screened for title and abstract, and 3252 articles were removed following our predefined study eligibility criteria. One hundred sixty-five articles underwent full-text screening for possible inclusion in the final qualitative analysis and data extraction, and 30 articles were included for qualitative analysis and data extraction. One hundred thirty-five articles were excluded due to the unavailability of the full article, some did not report any risk factor, mixed population (these articles reported the risk factors or population characteristics of different ethnic groups together), wrong population (these studies reported CRC together with other malignancies), not written in English language, case report, abstracts, comments, editorials, audit, and animal study. A full detail of the PRISMA flow diagram is shown in Figure 1.

Study Demographic

Our review included 53,596 people of African descent across nine countries (USA, Nigeria, Egypt, Morocco, Algeria, Zimbabwe, Tunisia, South Africa, and Uganda), and 8317 (16%) of them were diagnosed with CRC either via colonoscopy and histology. The USA contributed the most with 13 articles, Egypt and Morocco contributed four articles each, Zimbabwe, Uganda, and Tunisia contributed two articles each, and Nigeria, South Africa, and Algeria contributed one article each. The study period ranged from 1995 to 2023, and the mean age ranged from 34 to 64 years. Among those with CRC, 3198 (38%) were male, while 4130 (50%) were female. Table 1 below shows full details of the study demographics.

**Table 1:** Study demographics CRC: colorectal cancer; N/A: not available

Author	Country	Year	Population	Sample size	CRC	Mean age	Male	Female
Dash et al. [13]	USA	2009	Black women	3668	917	54	N/A	917
Williams et al. [14]	USA	2006	African American	384	225	58	52	173
Pibiri et al. [15]	USA	2006	African American	1799	961	63	523	438
Imad et al. [16]	Morocco	2017	African	450	225	55	119	106
Ashktorab et al. [17]	USA	2023	African American	58	23	60	11	12
Ashktorab et al. [18]	USA	2009	African American	280	93	59	49	44
Ochs-Balcom et al. [19]	USA	2014	African American	567	180	55	N/A	N/A
Satia-Abouta et al. [20]	USA	2000	African American	676	276	62	132	144
Zanetti et al. [21]	USA	2003	African American	103	45	64	N/A	N/A
Sansbury et al. [22]	USA	2000	African American	730	293	64	138	155
Katsidziraa et al. [23]	Zimbabwe	2012	African	300	100	53	50	50
Katsidziraa et al. [24]	Zimbabwe	2015	African	303	101	54	51	50
Kgomo et al. [25]	South Africa	2018	African	330	110	50	75	35
Lo et al. [26]	Egypt	2005	African	860	421	46	221	200
Kamal et al. [27]	Egypt	2019	African	200	100	46	46	54
Mahfouz et al. [28]	Egypt	2011	African	450	150	40	72	78
Gharbi et al. [29]	Tunisia	2019	African	100	51	56	27	24
Madbouly et al. [30]	Egypt	2001	African	60	60	34	38	22
Allam et al. [31]	Morocco	2018	African	120	70	60	45	25
Deoula et al. [32]	Morocco	2017	African	2906	1453	N/A	716	737
Barbirou et al. [33]	Tunisia	2018	African	600	300	56	146	154
El Asri et al. [34]	Morocco	2017	African	154	151	53	68	83
Wismayer et al. [35]	Uganda	2021	African	384	128	53	63	65
Wismayer et al. [36]	Uganda	2021	African	387	129	54	66	63
Negrichi et al. [37]	Algeria	2019	African	400	200	55	106	94
Ashktorab et al. [38]	USA	2012	African American	921	322	58	155	167
Brim et al. [39]	USA	2009	African American	1256	300	43	132	168
Barber et al. [40]	USA	2018	Black women	34421	204	39	N/A	N/A
Makambi et al. [41]	USA	1995	Black women	620	620	46	38	22
Agbo et al. [42]	Nigeria	2018	African	109	109	45	59	50

The largest sample size among the general population was 34,421; 1453 were among individuals with CRC, and the smallest sample size among the general population was 58; 23 were among individuals with CRC. The mean BMI ranged from 24 to 31 kg/m², with most studies reporting a BMI of 30 kg/m². The included study design is case control [13-37] and cohort studies [38-42]. 

Some of the included studies reported the site of the malignancy within the large intestine, and they were mainly located in the colon. The majority of the study participants are below a high/primary level of education among studies that reported participants' educational status. More details of the common site of the malignancy and educational qualification are shown in Tables 2-3.

**Table 2:** Tumor location

Large intestine	Number
Colon	2625
Rectum	741
Colorectal	11

**Table 3:** Educational qualification

Level of education	Number
Below high/primary school	1927
Some college/secondary school	590
Advanced degree/tertiary education	258

Risk and Protective Factors of CRC

There are many risk factors of CRC, which were highlighted by the various authors from the included studies. Some studies included the number of individuals at risk of CRC due to exposure to some factors, while the majority of the studies did not indicate this. We divided these factors into modifiable and non-modifiable factors; among the non-modifiable risk factors, age > 55 years, family history of CRC, male gender, family history of malignancy, and genetic mutation (single-nucleotide polymorphism (SNP), KRAS, p53, linkage disequilibrium, and klotho) were the most reported risk factors across the included studies. The modifiable risk factors include tobacco smoking, frequent consumption of alcohol, red/processed/roasted meat (suya), a fat diet, BMI > 30 kg/m², *Helicobacter pylori* infection, carcinogen exposure at work, and urban residence.

Among the protective factors against CRC, frequent consumption of vegetables, fruits, fish, traditional African diet, NSAID use, and physical activity were mostly reported. Details are shown in Table 4 below.

**Table 4:** Risk and protective factors of colorectal cancer BMI: body mass index; CRC: colorectal cancer; IGF: insulin-like growth factor; N/A: not available; NSAID: nonsteroidal anti-inflammatory drug; SNP: single nucleotide polymorphism

Modifiable factor	Non-modifiable factor	Protective factor
Tobacco smoking	Age > 55 years	High vegetable consumption
Frequent alcohol intake	Diabetes mellitus	Frequent fruit consumption
Frequent red/processed/roasted meat consumption	Single-nucleotide polymorphism	Deceased rice consumption
Frequent coffee consumption	Family history of CRC	Physical activity
High cake intake	Male gender	High fish consumption
High pasta intake	Chronic gastritis	NSAID use
High cream pastry intake	IGF-1	Traditional African dietary pattern
BMI > 30 kg/m²	Linkage disequilibrium	Increased parity
Low concentration of 25(OH)D3	Klotho haplotype variants mutation	Increased lactation duration
H. pylori infection	P53 overexpression in schistosome-associated CRC	N/A
Night shift job > 10 years	SNP on AKTI rs 10138227 genotype	N/A
High dietary Fat intake	SNP on the KCNIB gene	N/A
Urban residence > 1 year	KRAS mutation	N/A
History of schistosomiasis	Family history of GI, FAP, angiosarcoma, ulcerative colitis, carcinoid tumor.	N/A
Higher level of education	N/A	N/A
Non-NSAID intake for over five years	N/A	N/A
Frequent carcinogen/pesticide exposure	N/A	N/A
Daily consumption of spicy food	N/A	N/A
Daily soft drink/artificial sweetener intake	N/A	N/A
Frequent fast food consumption	N/A	N/A
Highly prudent diet	N/A	N/A
Fried/roasted/boiled chicken	N/A	N/A
High cheese intake	N/A	N/A

Pooled Prevalence of Colorectal Cancer

A meta-analysis was conducted to estimate the overall prevalence of CRC among individuals of African descent using data from 30 studies that reported both the sample size and the number of CRC cases. These studies, drawn from nine countries including the USA, Nigeria, Egypt, Morocco, Algeria, Zimbabwe, Tunisia, South Africa, and Uganda, encompassed a combined population of 53,596 individuals. Given the diversity in study settings and populations, a random-effects model using the DerSimonian-Laird method was employed to account for potential heterogeneity in effect sizes.

**Figure 2:**
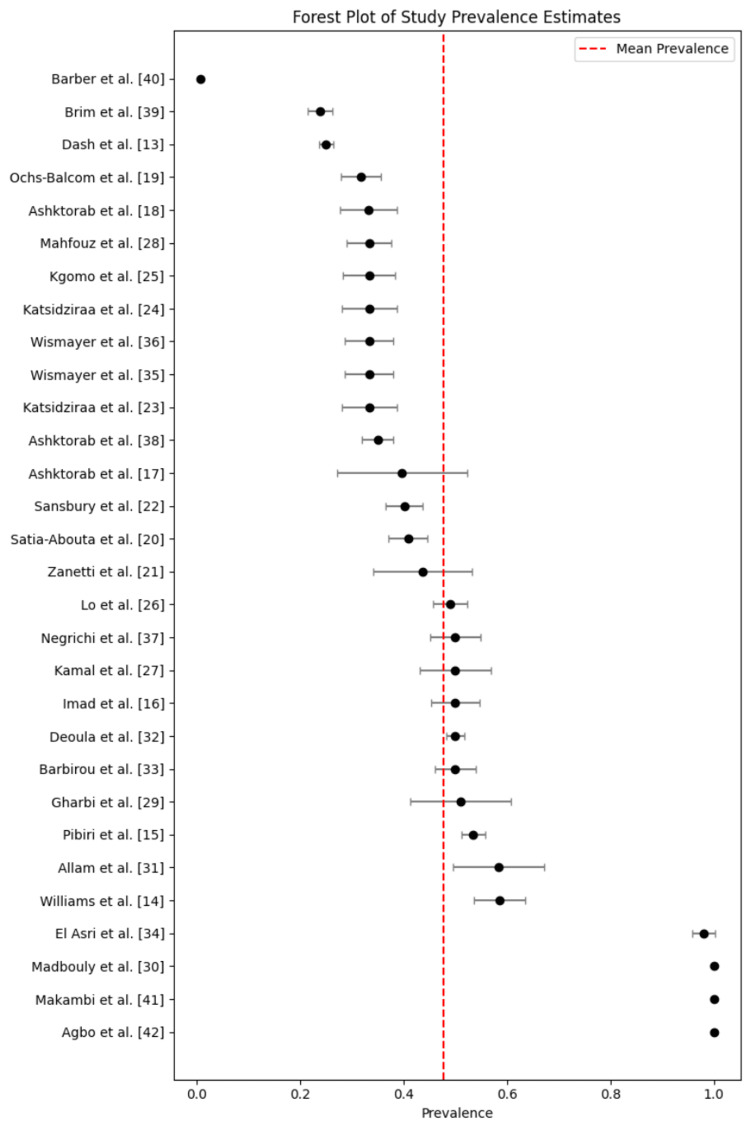
Forest plot showing the individual and pooled prevalence estimates of colorectal cancer among populations of African descent across 35 studies Horizontal lines represent 95% confidence intervals. The red vertical line indicates the pooled prevalence estimate derived using a random-effects model.

The pooled prevalence of CRC across the included studies was estimated at 41.8%, with a 95% confidence interval ranging from 39.6% to 44.1%. This indicates that, on average, more than four in ten individuals in the included populations had been diagnosed with CRC, either through colonoscopy or histological confirmation. This prevalence rate is notably higher than estimates reported in general screening populations globally, and it emphasizes the substantial burden of CRC among African and African-descended communities. A forest plot illustrating study-specific prevalence estimates and the pooled summary effect is shown in Figure 2.

Assessment of Heterogeneity

Despite the clarity of the pooled estimate, there was substantial heterogeneity observed across the included studies. Cochran’s Q statistic exceeded 1000, strongly suggesting that the variability among prevalence estimates was not due to chance alone. Furthermore, the I² statistic was above 98%, indicating that nearly all of the variation in effect size was attributable to real differences among studies, such as in design, population characteristics, diagnostic methods, or geographic and healthcare contexts. Additionally, the between-study variance, represented by Tau², was found to be non-zero, further reinforcing the presence of significant true variability in the reported CRC prevalence.

Synthesis of Risk Factors

In addition to the pooled prevalence, a qualitative synthesis of CRC risk factors was conducted based on their frequency of reporting across the included studies. This analysis identified both non-modifiable and modifiable risk factors that were commonly cited. Family history of malignancy emerged as the most frequently reported risk factor, mentioned in eight of the studies. Tobacco smoking followed closely, appearing in seven studies, while alcohol consumption and red meat intake were each cited in five. Obesity, typically defined as a BMI greater than 30 kg/m², was noted in four studies. Older age, particularly over 55 years, was another recurrent theme.

Genetic predispositions, including KRAS mutations and specific SNPs, were reported in six studies, underscoring the potential contribution of hereditary mechanisms in CRC pathogenesis among African descent populations. Other lifestyle-related risks included the frequent consumption of processed or high-fat meats and urban residence, both of which may reflect broader socioeconomic and dietary shifts occurring in these communities. A qualitative synthesis of reported risk factors across the included studies is summarized in Table 5.

**Table 5:** Most frequently reported colorectal cancer risk factors across 30 included studies, categorized by number of studies in which each factor appeared Risk factors are based on frequency of narrative or quantitative reporting within individual studies. BMI: body mass index; SNP: single-nucleotide polymorphism

Risk factor	Number of studies reporting
Family history of malignancy	8
Tobacco smoking	7
Alcohol consumption	5
Red meat consumption	5
Obesity (BMI > 30 kg/m²)	4
Genetic mutations (e.g., KRAS, SNPs)	6
Age > 55 years / older age	3
Processed or high-fat meat intake	3
Urban residence	2

Exploratory Subgroup Patterns

Although the data extracted from the studies were not sufficiently standardized to support formal meta-regression, an exploratory review of subgroup data revealed noteworthy patterns. Studies that identified current or former smoking status and positive family history of CRC tended to report higher prevalence estimates. Similarly, higher CRC prevalence was frequently observed in cohorts with elevated obesity rates or those reporting regular consumption of red or processed meat.

These findings, while not statistically tested through subgroup analysis, suggest that the interplay between genetic and lifestyle factors contributes significantly to CRC risk in these populations. They also point to the importance of detailed and structured subgroup reporting in future studies to enable more robust secondary analyses.

Discussion

This systematic review represents one of the most comprehensive efforts to explore risk and protective factors for CRC among populations of African descent, encompassing data from over 53,000 individuals across nine countries. Our findings offer significant insights, not only into the epidemiological patterns but also into sociocultural and genetic contributors to CRC risk in these historically underserved populations. This discussion contextualizes our results within existing literature and highlights their implications for research, clinical practice, and public health strategies.

Pooled Prevalence and Heterogeneity

The pooled prevalence (41.8%) observed in this study (Figure 2) highlights a markedly higher disease burden compared to global averages. As shown in Table 2, the most commonly identified risk factors include both genetic and modifiable lifestyle exposures, with smoking, obesity, and alcohol use consistently associated with higher CRC prevalence.

This value indicates a substantial burden, significantly higher than global averages, particularly in Western Europe and Asia, where pooled prevalence typically ranges between 10% and 25% in screening populations [43,44].

However, Cochran's Q statistic exceeded 1000, and the I² statistic surpassed 98%, indicating significant heterogeneity across studies. A non-zero τ² further suggests true variance in effect sizes between studies. These results imply that while CRC prevalence is consistently high among African-descent populations, regional differences, methodological variability, and population-specific risk profiles contribute substantially to this heterogeneity.

The elevated pooled prevalence should be interpreted in light of the fact that most included studies were hospital-based and sampled individuals already at higher risk due to symptoms or family history. These settings are not reflective of general population screening, which likely results in a lower true prevalence. Moreover, the substantial heterogeneity observed (I² > 98%) is attributable to variability in study design, diagnostic methods, population characteristics, and geographic regions.

Also, the pooled estimate must be interpreted with caution due to limited data from several Sub-Saharan African countries. While US-based diaspora studies are essential for understanding genetic and lifestyle transitions, their health systems, access to care, and environmental exposures differ markedly from many African settings. This imbalance underscores the urgent need for primary data generation in underrepresented regions to support culturally and regionally tailored CRC interventions.

Study Characteristics and Participant Distribution

The preponderance of data derived from US-based studies (n = 13) emphasizes the availability and accessibility of data in African-American populations, likely influenced by the structured health surveillance systems in place. This contrasts sharply with underrepresentation from sub-Saharan African countries, which, as several reviews point out, suffer from limited cancer registries and underdiagnosis [45,46]. Notably, studies from countries like Nigeria and Algeria accounted for only one article each despite their large populations. This geographic imbalance reinforces calls for expanding surveillance systems and research funding in African nations [47].

Sex and Age Disparities in CRC

We observed a higher proportion of CRC cases in women (50%) compared to men (38%). This deviates from global data, which typically reports a higher incidence among men (46). This may reflect gender-specific participation, sampling, or reporting biases in some population studies or sociocultural factors influencing screening behaviors among African men [48,49].

Age > 55 years emerged as the most consistent non-modifiable risk factor. This finding aligns with data from the African American Colorectal Cancer Study and broader epidemiological analyses, which show CRC risk increases significantly after age 50 [43,50]. However, emerging evidence also points to a rise in early-onset CRC (before age 50), which was not widely represented in the included studies [50].

Modifiable Risk Factors and Lifestyle Contributions

The most frequently reported modifiable risk factors - tobacco smoking, alcohol intake, high intake of processed meat, high-fat diets, and BMI >30 kg/m² - mirror established CRC risk profiles in Western cohorts [44,51]. These findings confirm a global epidemiological transition in dietary behaviors and lifestyle in urban African communities [52]. Urban residence, reported as a risk factor, likely proxies such lifestyle shifts.

Of particular interest is the strong association between *Helicobacter pylori* infection and CRC in several African studies. While *Helicobacter pylori* is well-documented in gastric cancer, its role in CRC has been explored with increasing frequency in African settings due to its high endemicity [45].

Occupational exposures (e.g., pesticide and carcinogen contact) and chronic gastritis also emerged in the literature and our analysis as key concerns in rural and industrial African settings, underscoring the environmental determinants of CRC [45].

Non-modifiable and Genetic Risk Factors

Several studies emphasized genetic mutations (e.g., KRAS, P53, and SNPs like AKT1 rs10138227), echoing molecular insights into CRC pathogenesis. These findings are consistent with evidence suggesting unique mutation profiles among African Americans and Africans, which differ from Caucasian populations and may impact screening and treatment responses [53].

Family history of CRC and related malignancies was one of the strongest predictive factors, reinforcing the importance of family history assessments in risk stratification protocols [54].

Protective Factors and Dietary Considerations

Protective behaviors, such as high vegetable and fruit intake, consumption of traditional African diets, and physical activity, were consistent across studies. These findings support broader nutritional epidemiology, including the World Cancer Research Fund (WCRF)/American Institute for Cancer Research (AICR) reports and recent dietary meta-analyses [44].

Interestingly, NSAID use was also reported as protective-a finding substantiated by multiple meta-analyses suggesting a role in chemoprevention [55]. However, long-term NSAID use poses gastrointestinal risks and must be balanced against benefits.

Educational attainment appeared as a protective factor in some studies, reflecting broader links between education and health-seeking behaviors, particularly screening uptake [46].

Strengths and limitations

Strengths

This study represents one of the most comprehensive systematic reviews and meta-analyses examining the prevalence and risk factors of CRC among populations of African descent. A major strength lies in the breadth and scope of the review, which synthesized data from 30 studies encompassing more than 53,000 individuals across nine countries, including both African and African-American populations. This wide representation allowed for a transcontinental perspective on the burden of CRC, facilitating comparative analysis across diverse sociocultural and health system contexts.

The methodological rigor of the study is also notable. The use of a PRISMA-compliant search strategy, adherence to predefined inclusion and exclusion criteria, and clear documentation of study selection ensure transparency and reproducibility. Furthermore, the application of a random-effects meta-analysis model (DerSimonian-Laird) allowed for the accommodation of substantial between-study heterogeneity, which was statistically confirmed through Cochran’s Q, I², and τ² values.

Another strength lies in the extraction and synthesis of both modifiable and non-modifiable risk factors. By parsing and analyzing both qualitative and quantitative data, the study offers a multidimensional understanding of CRC etiology in African descent populations, with implications for both clinical risk stratification and population-based prevention strategies.

Limitations

Despite its strengths, this study has several limitations that must be acknowledged. First, there was considerable heterogeneity across the included studies (I² > 98%), which reflects variation in study design, diagnostic criteria, population demographics, and data collection methods. While this heterogeneity was statistically accounted for, it limits the interpretability of the pooled prevalence as a singular representative metric.

Second, the quality and completeness of data reporting varied significantly across studies. Many studies did not provide disaggregated data by sex, age, or key covariates such as socioeconomic status or comorbidity profiles. Moreover, the reporting of risk factors was often narrative rather than numerical, which limited the ability to perform meta-regression or subgroup analyses with statistical rigor.

Third, the review was constrained by language bias, as only English-language studies were included. This may have led to the exclusion of relevant studies published in French, Arabic, Portuguese, or other languages spoken in Africa.

Lastly, genetic data and molecular profiling were inconsistently reported, making it difficult to draw robust conclusions about the role of specific genetic variants, such as KRAS or p53, despite their biological plausibility.

Recommendations and implications for practice

Recommendations for Research and Policy

Standardization of reporting: Future epidemiological studies on CRC in African populations should adopt standardized reporting formats, particularly for risk factors and diagnostic definitions, to facilitate meta-analytical synthesis and cross-study comparisons.

Expand geographic representation: There is a pressing need for prospective, registry-linked studies from underrepresented African countries. National cancer registries should be strengthened to provide longitudinal data on incidence, survival, and treatment outcomes.

Include molecular epidemiology: More research is needed to explore genetic and molecular risk markers, especially as precision medicine becomes more integrated into oncology care. Studies should aim to characterize mutational profiles specific to African ancestry.

Invest in community-based screening research: There should be investment in implementation science that explores culturally adapted screening interventions, including fecal occult blood testing, colonoscopy access, and mobile endoscopy clinics.

Clinical and Public Health Implications

The high pooled prevalence of CRC among African descent populations, along with the identified risk factor profile, has significant implications for both clinical practice and public health programming.

Screening guidelines: Current CRC screening guidelines may not adequately address the elevated risk in African descent populations. Lowering the age of initial screening to 40-45 years [56], especially for those with family history or metabolic risk factors, should be strongly considered.

Targeted health promotion: Health promotion campaigns should focus on modifiable risk factors such as tobacco use, obesity, diet, and physical inactivity. These campaigns must be culturally tailored and co-designed with community stakeholders.

Equity in access to care: The findings also highlight disparities in diagnostic access and healthcare infrastructure. Policy interventions are needed to reduce barriers to CRC screening in underserved African and African-American communities through subsidized screening programs, education, and infrastructure investment.

Genetic counseling and family risk assessment: With family history emerging as a dominant risk factor, clinicians should routinely collect three-generation cancer histories and refer high-risk individuals for genetic counseling, particularly in families with early-onset CRC or multiple malignancies.

## Conclusions

This systematic review and meta-analysis provide compelling evidence of a high burden of CRC among populations of African descent, with a pooled prevalence of approximately 41.8% across 35 studies. The substantial heterogeneity observed underscores the complex interplay of genetic, environmental, and lifestyle factors influencing CRC risk across diverse African and African-American populations.

Family history of malignancy, tobacco use, alcohol intake, obesity, and red or processed meat consumption emerged as the most consistently reported risk factors. Protective behaviors such as high vegetable intake, physical activity, and adherence to traditional African diets were underreported but suggest actionable avenues for prevention. The findings emphasize the urgent need for earlier and more culturally tailored screening interventions, as well as investment in molecular and registry-based research within African health systems.

## Appendices

Appendix A 

**Table 6:** Search strategy

Search query	Database	Outcome
((Risk factor) AND (colorectal cancer)) AND (African)	PubMed	684
((Risk factor) AND (colorectal cancer)) AND (African American)	PubMed	497
((Risk factor) AND (colorectal cancer)) AND (African Caribbean)	PubMed	12
((Risk factor) AND (colorectal cancer)) AND (Blacks)	PubMed	814
((Risk factor) AND (colorectal cancer)) AND (African European)	PubMed	56
"Colorectal cancer" "African Descent"	Google Scholar	1000
"Colorectal cancer" "African American"	Google Scholar	999
"Colorectal cancer" "Africa"	Google Scholar	1000

Appendix B

**Table 7:** Joanna Briggs Institute (JBI) critical appraisal for case-control studies

Checklist question	Dash et al. [13]	Williams et al. [14]	Pibiri et al. [15]	Imad et al. [16]	Ashktorab et al. [17]	Ashktorab et al. [18]	Ochs-Balcom et al. [19]	Satia-abouta et al. [20]	Zanetti et al. [21]	Sansbury et al. [22]	Katsidziraa et al. [23]	Katsidziraa et al. [24]	Kgomo et al. [25]	Lo et al. [26]	Kamal et al. [27]	Mahfouz et al. [28]	Gharbi et al. [29]	Madbouly et al. [30]	Allam et al. [31]	Deoula et al. [32]	Barbirou et al. [33]	El Asri et al. [34]	Wismayer et al. [35]	Wismayer et al. [36]	Negrichi et al. [37]
Were the groups comparable other than the presence of disease in cases or the absence of disease in controls?	Y	Y	Y	Y	Y	Y	Y	Y	Y	Y	Y	Y	Y	Y	Y	Y	Y	Y	Y	Y	Y	Y	Y	Y	Y
Were cases and controls matched appropriately?	Y	Y	Y	Y	Y	Y	Y	Y	Y	Y	Y	Y	Y	Y	Y	Y	Y	Y	Y	Y	Y	Y	Y	Y	Y
Were the same criteria used for identification of cases and controls?	Y	Y	Y	Y	Y	Y	Y	Y	Y	Y	Y	Y	Y	Y	Y	Y	Y	Y	Y	Y	Y	Y	Y	Y	Y
Was exposure measured in a standard, valid and reliable way?	Y	Y	Y	Y	Y	Y	Y	Y	Y	Y	Y	Y	Y	Y	Y	Y	Y	Y	Y	Y	Y	Y	Y	Y	Y
Was exposure measured in the same way for cases and controls?	Y	Y	Y	Y	Y	Y	Y	Y	Y	Y	Y	Y	Y	Y	Y	Y	Y	Y	Y	Y	Y	Y	Y	Y	Y
Were confounding factors identified?	Y	Y	Y	Y	Y	Y	Y	Y	Y	Y	Y	Y	Y	Y	Y	Y	Y	Y	Y	Y	Y	Y	Y	Y	Y
Were strategies to deal with confounding factors stated?	Y	Y	Y	Y	Y	Y	Y	Y	Y	Y	Y	Y	Y	Y	Y	Y	Y	Y	Y	Y	Y	Y	Y	Y	Y
Were outcomes assessed in a standard, valid, and reliable way for cases and controls?	Y	Y	Y	Y	Y	Y	Y	Y	Y	Y	Y	Y	Y	Y	Y	Y	Y	Y	Y	Y	Y	Y	Y	Y	Y
Was the exposure period of interest long enough to be meaningful?	Y	Y	Y	Y	Y	Y	Y	Y	Y	Y	Y	Y	Y	Y	Y	Y	Y	Y	Y	Y	Y	Y	Y	Y	Y
Was appropriate statistical analysis used?	Y	Y	Y	Y	Y	Y	Y	Y	Y	Y	Y	Y	Y	Y	Y	Y	Y	Y	Y	Y	Y	Y	Y	Y	Y

Appendix C

**Table 8:** Joanna Briggs Institute (JBI) critical appraisal for cohort studies

Checklist question	Ashktorab et al. [38]	Brim et al. [39]	Barber et al. [40]	Makambi et al. [41]	Agbo et al. [42]
Were the two groups similar and recruited from the same population?	Y	Y	Y	Y	Y
Were the exposures measured similarly to assign people to both exposed and unexposed groups?	Y	Y	Y	Y	Y
Was the exposure measured in a valid and reliable way?	Y	Y	Y	Y	Y
Were confounding factors identified?	Y	Y	Y	Y	Y
Were strategies to deal with confounding factors stated?	Y	Y	Y	Y	Y
Were the groups/participants free of the outcome at the start of the study (or at the moment of exposure)?	N	N	N	N	N
Were the outcomes measured in a valid and reliable way?	Y	Y	Y	Y	Y
Was the follow-up time reported and sufficient to be long enough for outcomes to occur?	N	N	N	N	N
Was follow-up complete, and if not, were the reasons for loss to follow-up described and explored?	N	N	N	N	N
Were strategies to address incomplete follow-up utilized?	N	N	N	N	N
Was appropriate statistical analysis used?	Y	Y	Y	Y	Y
